# Flexible 6-in-1 Microsensor for Real-Time Microscopic Monitoring of Proton Battery

**DOI:** 10.3390/membranes11080615

**Published:** 2021-08-12

**Authors:** Chi-Yuan Lee, Chia-Hung Chen, Chin-Yuan Yang, John-Shong Cheong, Yun-Hsiu Chien, Yi-Chuan Lin

**Affiliations:** 1 Yuan Ze Fuel Cell Center, Department of Mechanical Engineering, Yuan Ze University, Taoyuan 32003, Taiwan; roy6051010@gmail.com (C.-Y.Y.); johnshong1018@gmail.com (J.-S.C.); s1040923@g.yzu.edu.tw (Y.-H.C.); chuan881018@gmail.com (Y.-C.L.); 2 HOMYTECH Global CO., LTD., Taoyuan 33464, Taiwan; chenjahon@gmail.com

**Keywords:** proton battery, flexible 6-in-1 microsensor, real-time microscopic monitoring

## Abstract

According to the comparison between a proton battery and a proton exchange membrane fuel cell (PEMFC), the PEMFC requires oxygen and hydrogen for generating electricity, so a hydrogen tank is required, leading to larger volume of PEMFC. The proton battery can store hydrogen in the carbon layer, combined with the oxygen in the air to form water to generate electricity; thus, the battery cost and the space for a hydrogen tank can be reduced a lot, and it is used more extensively. As the proton battery is a new research area, multiple important physical quantities inside the proton battery should be further understood and monitored so as to enhance the performance of battery. The proton battery has the potential for practical applications, as well as water electrolysis, proton storage and discharge functions, and it can be produced without expensive metals. Therefore, in this study, we use micro-electro-mechanical systems (MEMS) technology to develop a diagnostic tool for the proton battery based on the developed microhydrogen sensor, integrated with the voltage, current, temperature, humidity and flow microsensors developed by this laboratory to complete a flexible integrated 6-in-1 microsensor, which is embedded in the proton battery to measure internal important physical parameters simultaneously so that the reaction condition in the proton battery can be mastered more accurately. In addition, the interaction of physical quantities of the proton battery are discussed so as to enhance the proton battery’s performance.

## 1. Introduction

The proton battery is a very novel research area. It was first proposed by Heidari et al. from the Royal Melbourne Institute of Technology (RMIT), Australia, in March 2018 [1]. It is rechargeable and can electrolyze water. The electrolyzed protons can be stored, and the protons can be released to combine with oxygen to generate electricity. The hydrogen storage material is activated carbon instead of expensive metals, so it is a lower cost and safe.

At present, the fuel cell technology has been used in different areas, but there are still some problems to be solved, such as size, weight and hydrogen storage problems. Folonari et al. [2,3] indicated that besides a higher energy density and a more compact and simple structure, the new kind of fuel cell composed of hydride electrode and solid electrolyte could maintain the advantages of a conventional fuel cell. This kind of structure could enhance the reliability and performance of electric vehicles significantly. In terms of the most mainstream fuel cells at present, the hydrogen is stored in the hydrogen storage tank, and the hydrogen storage tank is installed onto the carrier, such as vehicles and generators. Most hydrogen storage tanks use the metal hydride technique to store hydrogen. In terms of the unitized regenerative fuel cell using a metal hydride–nitride composite electrode, the storage volume of hydrogen in the electrode measured in charging mode was 0.6 wt%. The discharged hydrogen volume was detectable, but very low, only about 0.01 wt%, and there would be safety risk [4]. Jurewicz et al. [5,6,7] pyrolyzed viscose fabric of liquid KOH electrolyte (concentration 6 M) to prepare an activated carbon electrode for electrochemical hydrogen storage, implemented 1.5 wt% reversible hydrogen storage, and studied the influence of acidity and alkalinity of electrolytes on the hydrogen storage capacity. Babel et al. [8] fabricated a highly porous carbon; the 1.89 wt% high hydrogen storage capacity proved that high ultramicropore and micropore volume and relatively smaller mesopore volume enhanced the hydrogen storage capacity. Bosch et al. [9] enlarged the surface area and improved the material. As the nano-structure hydrogen storage characteristic of carbon was known, the feasibility of rechargeable proton battery with a high energy density and high hydrogen storage capacity could be increased. The proton battery is a very novel research area, and there are many immature prototypes [10,11,12,13]; therefore, it is a topic worthy of exploration.

The proton battery design of Andrews et al. [14] used a solid electrode made of a metal alloy to store hydrogen. The protons escape from the membrane, and the electrons combine with metal in the external circuit to form hydride.

Guterl and Fang [15,16] found in the highly ordered porous carbon material that the hydrogen storage capacity was linearly related to the volume of the ultramicropore. According to the document [17,18,19,20,21,22,23,24,25,26,27,28], it is difficult to measure multiple important physical parameters inside the proton battery accurately, and the multiple important physical parameters (e.g., hydrogen, voltage, current, temperature, humidity and flow) are correlated with each other; all of them have a critical effect on the performance of the proton battery. However, the external measurement of the proton battery is the only way at the moment to know the overall output performance, and the authentic information inside the proton battery cannot be known accurately and instantly. The internal diagnosis of the proton battery is a valuable research field. In this study, we use micro-electro-mechanical systems (MEMS) technology to develop a flexible integrated microsensor embedded into the proton battery to provide valuable data.

## 2. Process of Flexible 6-in-1 Microsensor

**Figure 1:**
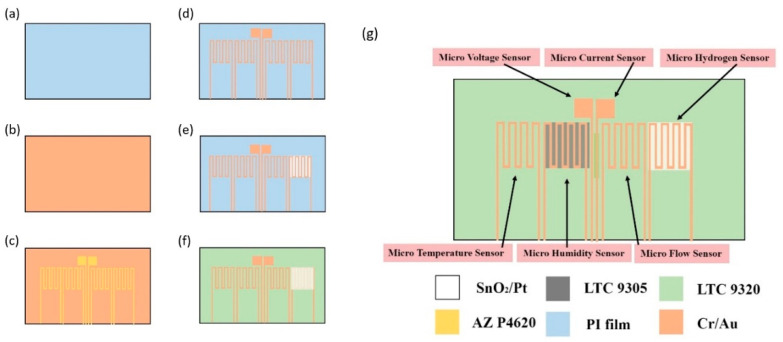
The process diagram of the flexible 6-in-1 microsensor.

This study used MEMS technology to integrate six sensing functions, including temperature, voltage, current, flow, humidity and hydrogen. The process of the flexible 6-in-1 microsensor is shown in Figure 1.

**Figure 2:**
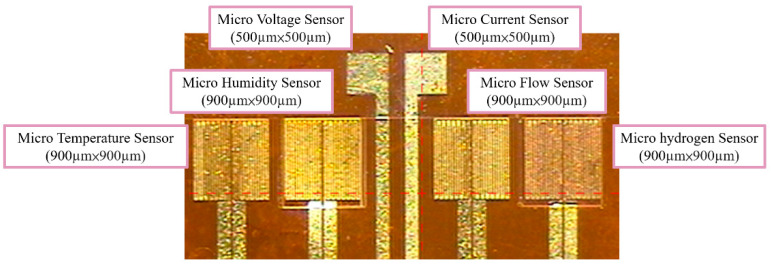
The optical micrograph of the flexible 6-in-1 microsensor.

**Figure 3:**
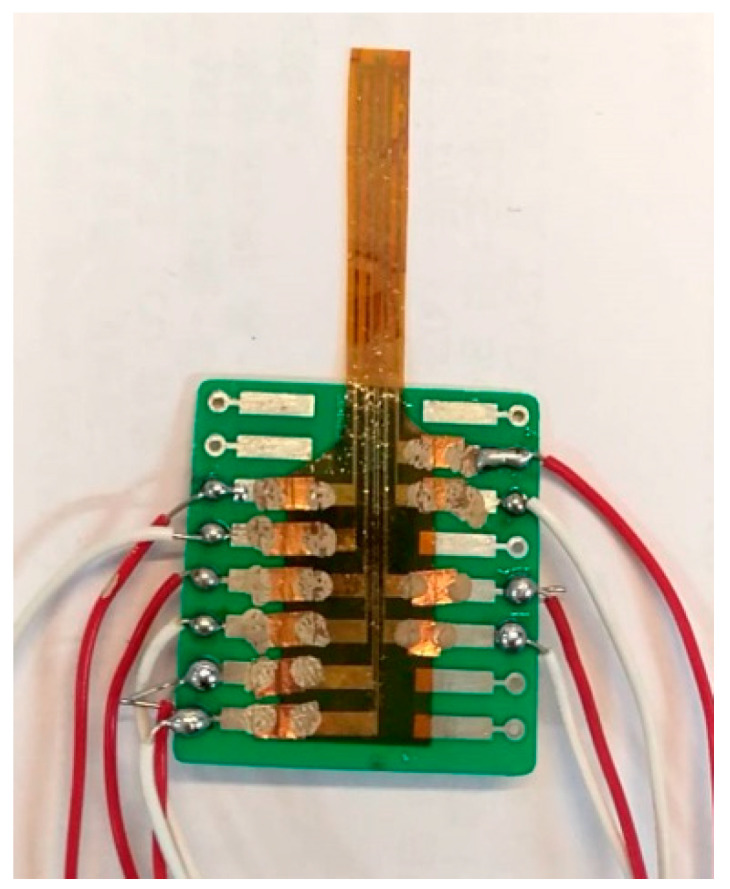
Stereogram of the flexible 6-in-1 microsensor.

(a) First, the PI film is cleaned with acetone and methanol organic solutions, respectively. The residual methanol is removed by DI water, and the surface dust and residual oil and fat are removed so as to enhance the adhesive ability of thin film metal.
(b) The Cr is evaporated by an E-beam evaporator as the adhesion layer of Au and PI film, the adhesiveness of Au and PI film is increased, and the deposition of 1000 Å thick Au is completed at a deposition rate of 0.1 Å/s.
(c) The AZ P4620 (positive photoresist) is spin coated, and the electrode pattern of the microsensor is defined by exposure and development.
(d) The Au is etched by Au etching solution (Type-TFA), and the Cr is etched by Cr etching solution (Cr-7T). The photoresist as an etching mask is removed by Remove 1165.
(e) The AZ P4620 is spin coated again as the mask for evaporation, and the microsensor pattern is defined by the exposure, and then the required pattern is developed by developer.
(f) The SnO_2_ is evaporated on the surface where the pattern has been defined as the gas sensing layer, and the Pt is evaporated as the catalyst layer.
(g) The sample is put in the 80 °C photoresist remover (Remove 1165) and kept still for about 20 min The photoresist mask is lifted off by using lift-off method, and then the sample is cleaned with acetone and methanol.
(h) Finally, the LTC 9320 (negative photoresist) is spin coated on the flexible 6-in-1 microsensor to complete the protection layer and fabrication. Figure 2 and Figure 3 are the optical micrograph and stereogram of the flexible 6-in-1 microsensor, respectively.

## 3. Correction of Flexible 6-in-1 Microsensor

The micro voltage and current sensors directly export the voltage and current from the proton battery for detection, additional correction is unnecessary. The correction of micro temperature, humidity, flow and hydrogen sensors is described below.

### 3.1. Temperature Distribution inside Water Electrolyzer

**Figure 4:**
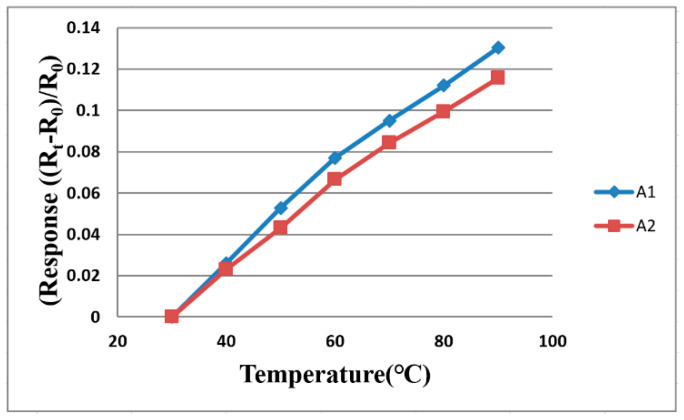
The correction curve of two micro temperature sensors.

The flexible 6-in-1 microsensor and the thermometer of BM-525 BRYMEN digital multimeter are put in a DENG YNG DS45 Drying Oven. After the reference control temperature is set up and stabilized, the resistance value of the micro temperature sensor is extracted. In the working temperature range, the resistance value is extracted at intervals of 10 °C, and the micro temperature sensor is corrected three times. The correction curve of two micro temperature sensors is measured. The curve approximates the linear variation, as shown in Figure 4.

### 3.2. Humidity Correction of Flexible 6-in-1 Microsensor

**Figure 5:**
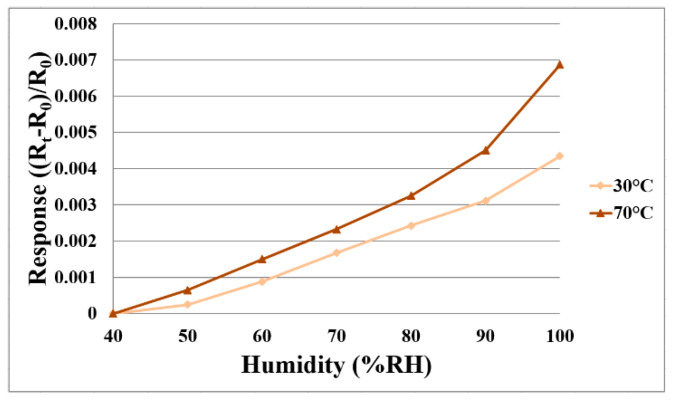
The correction curve of two micro humidity sensors.

For humidity correction, the constant temperature and humidity testing machine is used as environmental criteria from relative humidity 40% to 100%, and the temperatures (30 °C and 70 °C) are fixed in operating condition. Each time when the relative humidity recording point is increased, the heater on the micro humidity sensor is heated to evaporate the residual moisture at the previous recording point. Each time after 120 min of stabilization, the NI PXI 2575 data acquisition unit is used to extract the resistance value of the micro humidity sensor instantly, so as to obtain the correction curve. Figure 5 shows the correction curve of two micro humidity sensors. The higher the temperature is, the larger is the humidity variation.

### 3.3. Flow Correction of Flexible 6-in-1 Microsensor

**Figure 6:**
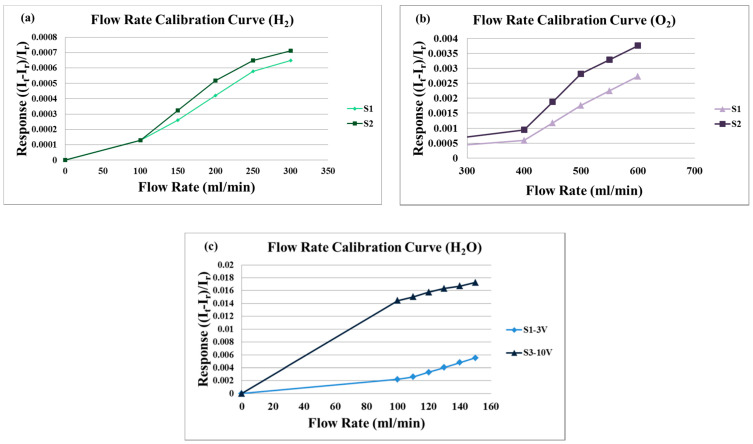
(**a**) The correction curve of two micro flow sensors (H_2_). (**b**) The correction curve of two micro flow sensors (O_2_). (**c**) The correction curve of two micro flow sensors (H_2_O).

As the proton battery is supplied with gases and water during charging and discharging, it is necessary to correct the water flow. The flexible 6-in-1 microsensor is embedded in the proton battery, the battery testing machine supplies gases (H_2_ and O_2_) as the source of flow. The power supply is connected to the signal pin of the micro flow sensor, and the anode is connected to BM-525 BRYMEN digital multimeter in a series to measure the current variation. The power supply supplies a constant voltage to the microflow sensor to generate a stable heat. First, the reference current value at 0 mL/min is measured. The hydrogen flow correcting range is 100 to 300 mL/min, and it is measured at intervals of 50 mL/min. The oxygen flow correcting range is 400 to 600 mL/min, and it is measured at intervals of 50 mL/min. The measured correction curve of two microflow sensors is shown in Figure 6a,b.

For liquid (H_2_O) flow correction, the LEADFLUID BT100S-1 speed adjusting peristaltic pump provides a steady flow. The flow correction range is 100 to 150 mL/min, measured at intervals of 10 mL/min. The correction curve of two micro flow sensors is shown in Figure 6c.

### 3.4. Hydrogen Correction of Flexible 6-in-1 Microsensor

**Figure 7:**
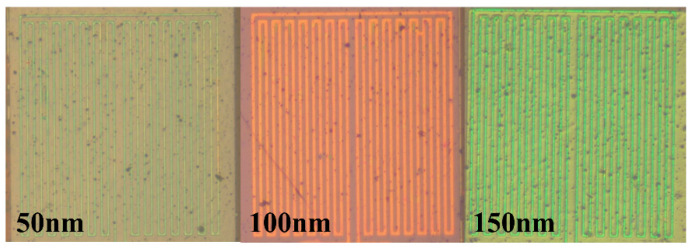
Optical micrograph of evaporated SnO_2_ in different thicknesses (50, 100, 150 nm) (2.5 nm thick Pt is evaporated on the uppermost layer as catalyst layer).

**Figure 8:**
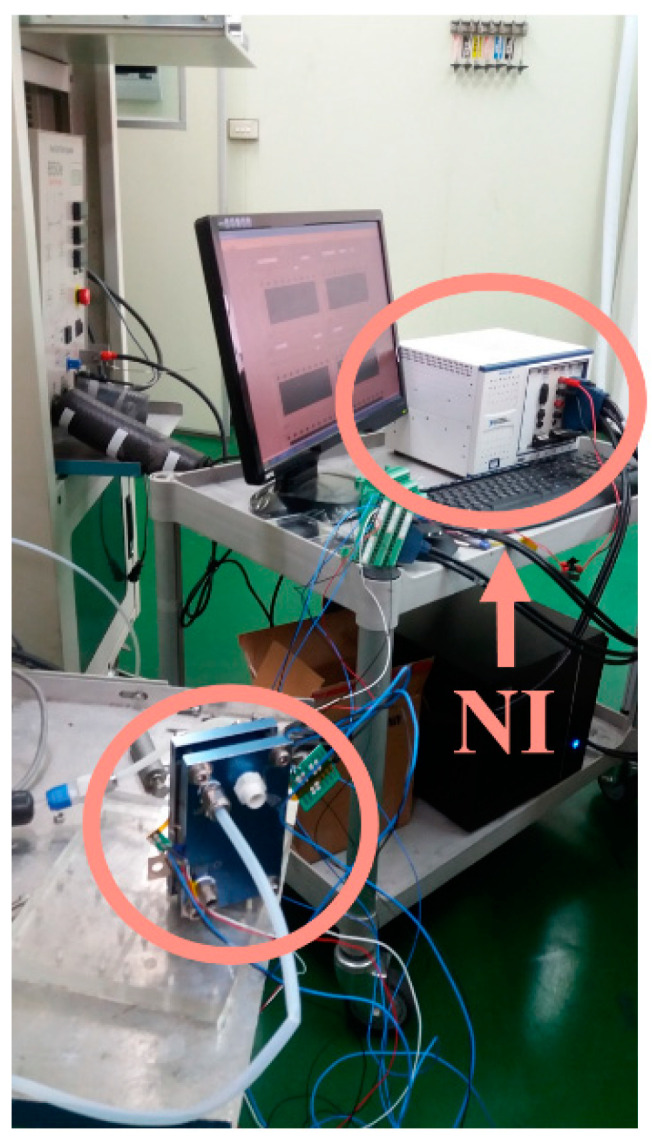
Test and correction of micro hydrogen sensor.

The flexible micro hydrogen sensor performance testing aims to know the sensing condition and efficiency of the micro hydrogen sensor and to determine the operation and process optimization parameters in order to manufacture a microhydrogen sensor operated at low temperatures. Figure 7 shows the optical micrograph of SnO_2_ in different thicknesses (50, 100, 150 nm) (the uppermost layer is coated with 2.5 nm thick Pt as catalyst layer). The micro hydrogen sensor is tested and corrected by a fuel cell testing machine, as shown in Figure 8. The fuel cell testing machine can supply different gases, set up the gas flow and heat gases. Therefore, the microhydrogen sensor is embedded in the proton battery runner plate, and the flow channel of proton battery is used as a closed chamber for the test. First, the micro hydrogen sensor is connected to NI PXI-1033 of a computer to detect the resistance change, supplied with oxygen at a constant temperature and constant flow so that the surface of micro hydrogen sensor can adsorb oxygen ions (O^−^). Afterwards, the hydrogen at a constant temperature and constant flow is supplied, and the O^−^ is carried away from the surface of micro hydrogen sensor so that the micro hydrogen sensor resistance decreases. The hydrogen is detected using the resistance difference among different gases.

## 4. Internal Real-Time Microscopic Diagnosis of Proton Battery

The flexible 6-in-1 microsensor can be embedded in the proton battery before the experiment. The activated carbon powder is put in the hydrogen-side flow channel before assembly. After assembly, the sulfuric acid is added to the hydrogen side through the upstream inlet. The flexible 6-in-1 microsensor is used for internal data and proton battery analysis, real-time microscopic monitoring of changes in temperature, voltage, current, flow and humidity and detecting the generation of hydrogen.

**Figure 9:**
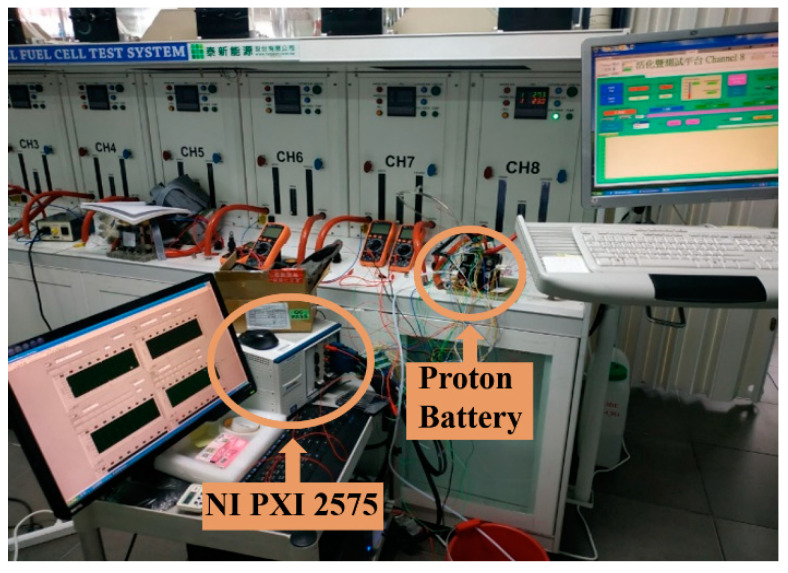
Discharge test.

The fuel cell testing equipment is used for the discharge test. The pure oxygen is added to the oxygen side at room temperature (25 °C), the flow is set as 500 mL/min, as shown in Figure 9.

### 4.1. Internal Runner Design for Proton Battery

The main purpose of runner design is to uniformly distribute fluid over the proton exchange membrane. The runner design of the bipolar plate is related to the proton battery efficiency, so the area, width and depth of the runner can influence the reaction of the proton battery.

**Figure 10:**
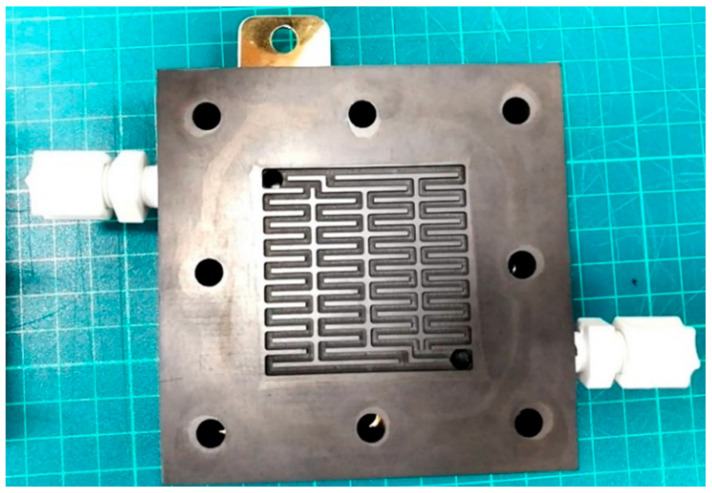
Serpentine nonparallel runner plate1.

The common designs of runner plate include mesh flow field, parallel flow field, single serpentine flow field and double serpentine flow field. The selected runner design is a serpentine nonparallel flow field (area is 25 cm^2^), as shown in Figure 10. Table 1 shows the bipolar runner plate specification.

### 4.2. Proton Battery Assembly Method

**Figure 11:**
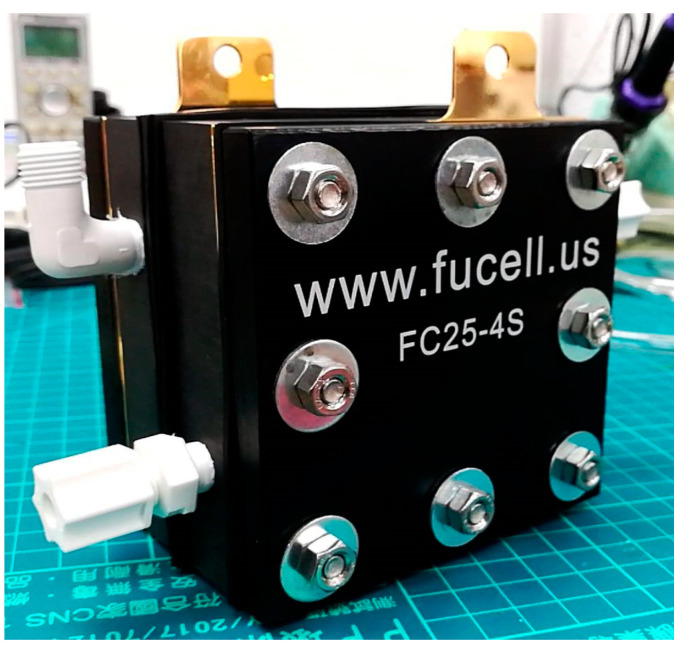
The proton battery.

Assembly process:

(a) Remove dirt with ethanol and DI water;
(b) Cover the bolt with thermal shrinkage agent to prevent short circuit of battery;
(c) Fix four bolts into the holes in the end plate for positioning and subsequent battery assembly;
(d) Fix the silicon spacer between the end plate and collector plate by bolt so that the silicon spacer can be pressed uniformly when fixing the battery;
(e) Place the collector plate on the silicon resin washer using bolt;
(f) Place the bipolar plate with runner up above the collector plate;
(g) Place the MEA above the bipolar plate;
(h) Place another bipolar plate on the MEA, the runner faces the MEA;
(i) Place on another collector plate;
(j) Place on the silicone rubber gasket;
(k) Place on another end plate;
(l) Diagonally fix the nut to 10 kgf·cm with a torque wrench. The torque of each nut must be the same and increased by 5 kgf·cm till 25 kgf·cm, so as to prevent the bipolar plate and collector plate from deforming or breaking due to nonuniform stress during tightening, the clamping pressure too small will also cause the gas diffusion layer unable to contact the graphite plate and make the battery unable to charge normally;
(m) The proton battery assembly is completed, as shown in Figure 11.

### 4.3. Calibration of Flexible Iintergrated 6-in-1 Microsensor and Proton Battery

**Figure 12:**
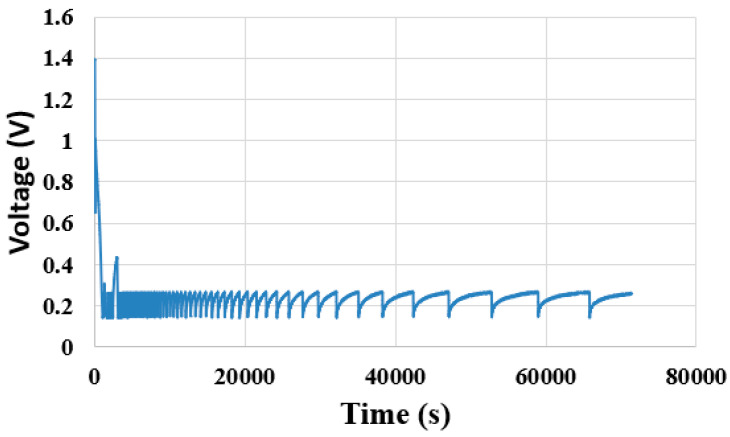
Voltage changes during the proton battery discharge.

**Figure 13:**
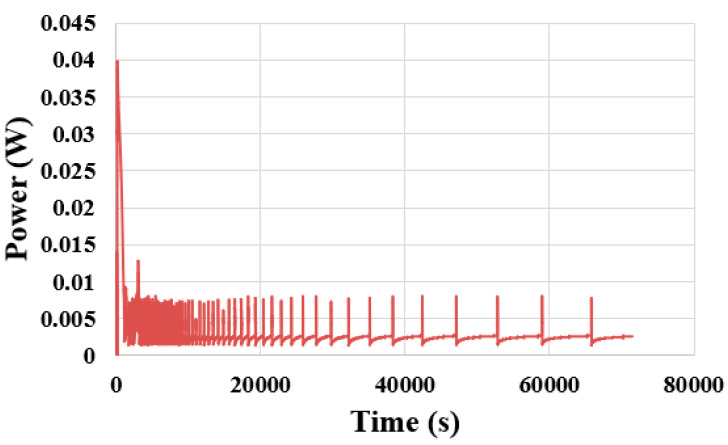
Power output during the proton battery discharge.

The constant current is adjusted to 40 mA according to the performance of the proton battery, and the changes in voltage, current and power are observed, as shown in Figure 12 and Figure 13. The discharging efficiency of the proton battery is about 0.37 wt%, and the volume of released hydrogen is only half of the volume of the absorbed hydrogen. This may be related to the catalyst layer of the proton exchange membrane.

### 4.4. Temperature Distribution inside Proton Battery

**Figure 14:**
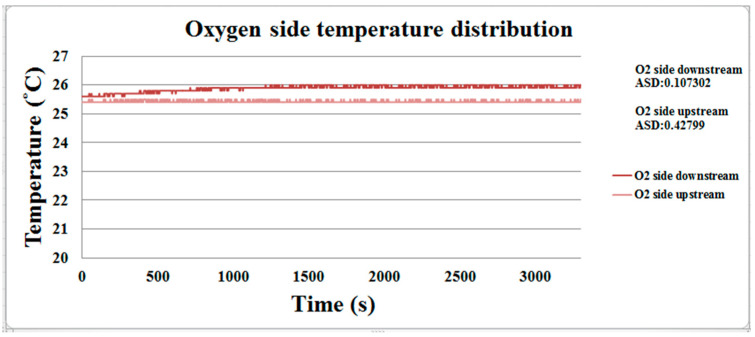
The oxygen side’s temperature distribution during the proton battery discharge.

**Figure 15:**
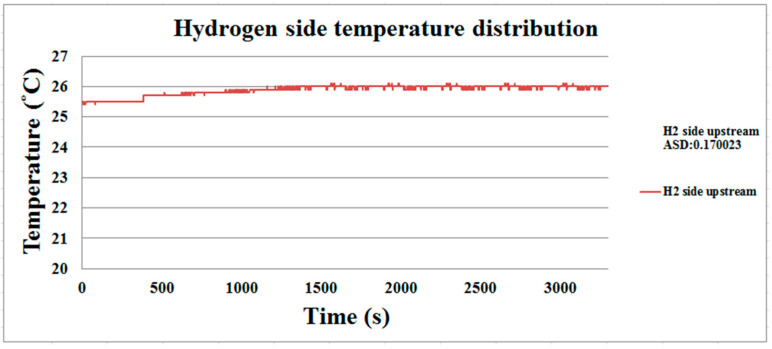
The hydrogen side’s temperature distribution during the proton battery discharge.

The discharge process of the proton battery is performed at room temperature (25 °C), and the temperature distribution is shown in Figure 14 and Figure 15. The downstream oxygen side temperature is higher than the upstream temperature. Due to the reaction, the upstream temperature of the hydrogen side will rise slightly, and then the temperature is stabilized at about 26 °C as the release efficiency decreases.

### 4.5. Flow Distribution inside Proton Battery

**Figure 16:**
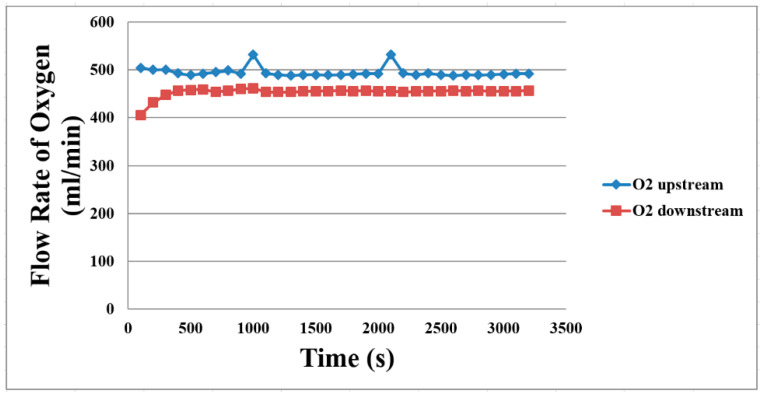
The oxygen side’s flow distribution during the proton battery discharge.

The flow velocity set in the fuel cell testing equipment is 500 mL/min, as shown in Figure 16. The upstream flow is higher than the downstream flow, which resulted from the power generation reaction and gas diffusion layer. In the initial stage, the downstream flow velocity is lower, meaning the earlier the power generation reaction is, the higher is the gas flow, and then when the reaction slows down, the flow velocity becomes stable.

### 4.6. Humidity Distribution inside Proton Battery

**Figure 17:**
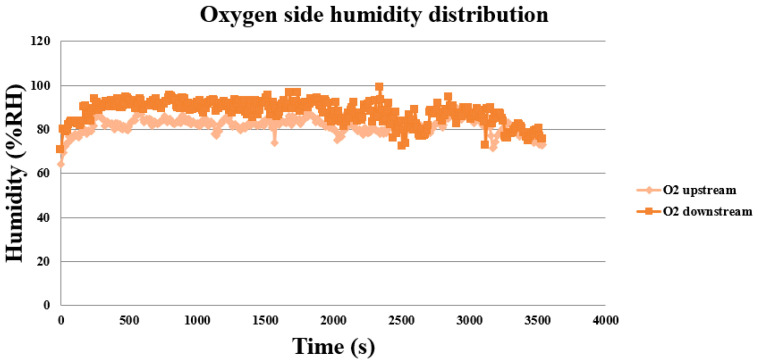
The oxygen side’s humidity distribution during the proton battery discharge.

The fuel cell testing equipment supplies wet oxygen so that the proton exchange membrane has a good working environment and the measured humidity is higher. As shown in Figure 17, the oxygen side increases slowly from lower humidity, and the downstream humidity is higher. This is related to the combination of hydrogen and oxygen. However, as the electricity generation decreases, the reaction is very slight, and the upstream and downstream humidities are overlapped in the late stage.

### 4.7. Hydrogen Distribution inside Proton Battery

**Figure 18:**
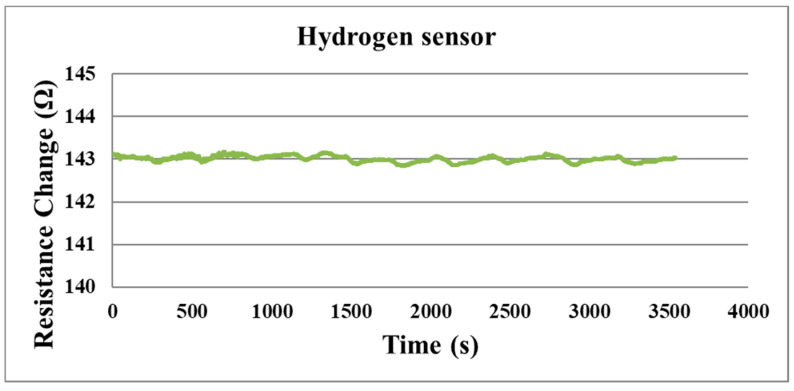
The hydrogen side’s hydrogen detection.

The resistance changes measured by the micro hydrogen sensor on the hydrogen side are shown in Figure 18. Since the electrical group of the hydrogen sensor is very small, the ohmic change is not obvious, so it can also be known that the micro hydrogen sensor needs oxygen for restoring the conditions for detecting hydrogen. The micro hydrogen sensor cannot detect hydrogen very well due to the hydrogen side is sealed.

### 4.8. Discussion

The operating temperature was set to 25 °C, and the microsensor was placed in different places in the oxygen side and the hydrogen side to observe the temperature distribution in the proton battery. From the oxygen side, it can be found that the water inlet is upstream, so the upstream temperature is lower, and the downstream temperature higher is because the temperature will increase after the reaction. Since sulfuric acid does not flow on the hydrogen side, the battery will tend to heat up gradually. On the oxygen side, the flow rate of water distribution upstream has a larger flow rate than downstream due to the water electrolysis inside the proton battery, and GDL could lead to resistance of the water flow. On the hydrogen side, we only need to measure the hydrogen flow rate, but the measured value is very strange. It is because the change of current of the micro flow sensor is too small and exceeds the limit of the measuring equipment we use. On the other hand, the flow rate of hydrogen at the hydrogen side is incoherent and makes the heated micro flow sensor unable to measure accurately. The measurement of the miniature hydrogen sensor cannot work normally in a closed space. In addition, the micro flow sensor cannot be measured in a non-constant current state based on the limitation of the measuring device, so the measurement results of these two physical quantities are not considered successful.

## 5. Conclusions

In this study, the micro temperature, voltage, current, flow, humidity and hydrogen sensors were successfully integrated onto a 50 μm thick PI film using MEMS technology. This flexible 6-in-1 microsensor has six sensing structures. It is characterized by a small thickness, small area, high sensitivity and arbitrary placement. The flexible 6-in-1 microsensor is embedded in the runner plates of the oxygen side and hydrogen side of the proton battery. The proton battery sealing condition was not influenced. In the operation of the proton battery, the changes in the temperature, voltage, current, flow, humidity and hydrogen of the proton battery were measured successfully. During the discharge of the proton battery, the power output of only 0.37 wt% is obtained. The amount of hydrogen released is only half of the amount of hydrogen absorbed, which may be related to the catalytic layer of the proton exchange membrane, which is more suitable for a water electrolysis reaction but not suitable for a reverse reaction. Future research should be aimed at miniaturizing the microsensor, which can reduce the effect of the microsensor on the proton battery. In the proton battery part, the hydrogen storage-activated carbon can move to the outside of the battery, and the activated carbon and sulfuric acid should be circulated in the battery through the pump, which can remove the restriction of the proton battery on the amount of hydrogen storage material. Then, find a better catalyst layer of the proton exchange membrane, which can provide better performance when in the charge mode or discharge mode.

## Figures and Tables

**Table 1:** Specification of the bipolar runner plate.

Characteristic	Specification
Thickness of BP (mm)	20
Channel type	Multi-serpentine flow field
Channel depth (mm)	1.5
Channel width (mm)	1.5
Rib width (mm)	1
